# Outpatient superficial partial‐thickness burn care of an elderly patient successfully treated with Eppikajutsuto

**DOI:** 10.1002/jgf2.685

**Published:** 2024-03-12

**Authors:** Hiroki Ishibashi, Ryo Yoshinaga, Tetsuhisa Yamada

**Affiliations:** ^1^ Department of Emergency Iizuka Hospital Iizuka City Japan; ^2^ Munakata City National Health Insurance Oshima Clinic Munakata City Japan; ^3^ Department of Japanese Oriental Medicine Iizuka Hospital Iizuka City Japan

**Keywords:** Eppikajutsuto, family medicine, geriatrics, second‐degree burn injury, superficial partial‐thickness burn

## Abstract

Eppikajutsuto (EPTJ) is used to reduce redundant body fluids and suppress inflammation. We observed that EPTJ shortened the duration of treatment in an elderly patient with burn injuries. A 96‐year‐old man suffered superficial partial‐thickness burns on the dorsum of his right hand and left knee. The injuries showed early improvement with the use of EPTJ. This suggests that EPTJ could contribute to shortening the duration of healing for superficial partial‐thickness burns in elderly patients who may experience slow wound healing and have a high mortality rate. Eppikajutsuto needs to be considered as a treatment for burns in primary care.

## Graphical abstract

We observed that EPTJ shortened the duration of treatment in a 96‐year‐old man with superficial partial‐thickness burn injuries. Eppikajutsuto needs to be considered as a treatment for burns in primary care.

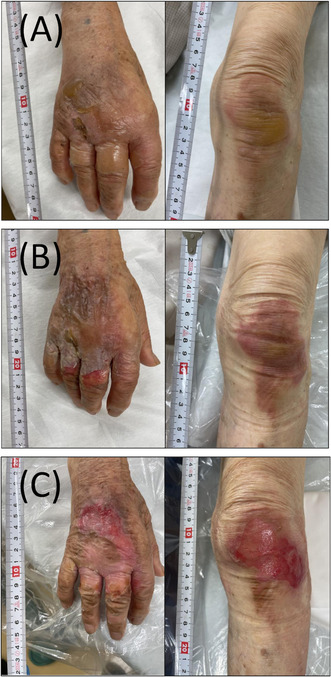

## 1 INTRODUCTION

Kampo, traditional Japanese herbal medicine, is frequently used treatments in primary care. Eppikajutsuto (EPTJ) is employed to reduce redundant body fluids and suppress inflammation; it is known for its efficacy in treating rheumatoid arthritis and eczema. It has been shown to be effective for atopic dermatitis.^1^ Despite the fact that the pathophysiology of burn injuries involves both inflammation and tissue edema, there have been no previous reports of EPTJ being used for burns. We encountered a case where the duration of treatment was reduced using EPTJ in an elderly burn patient.

## 2 CASE

**FIGURE 1:**
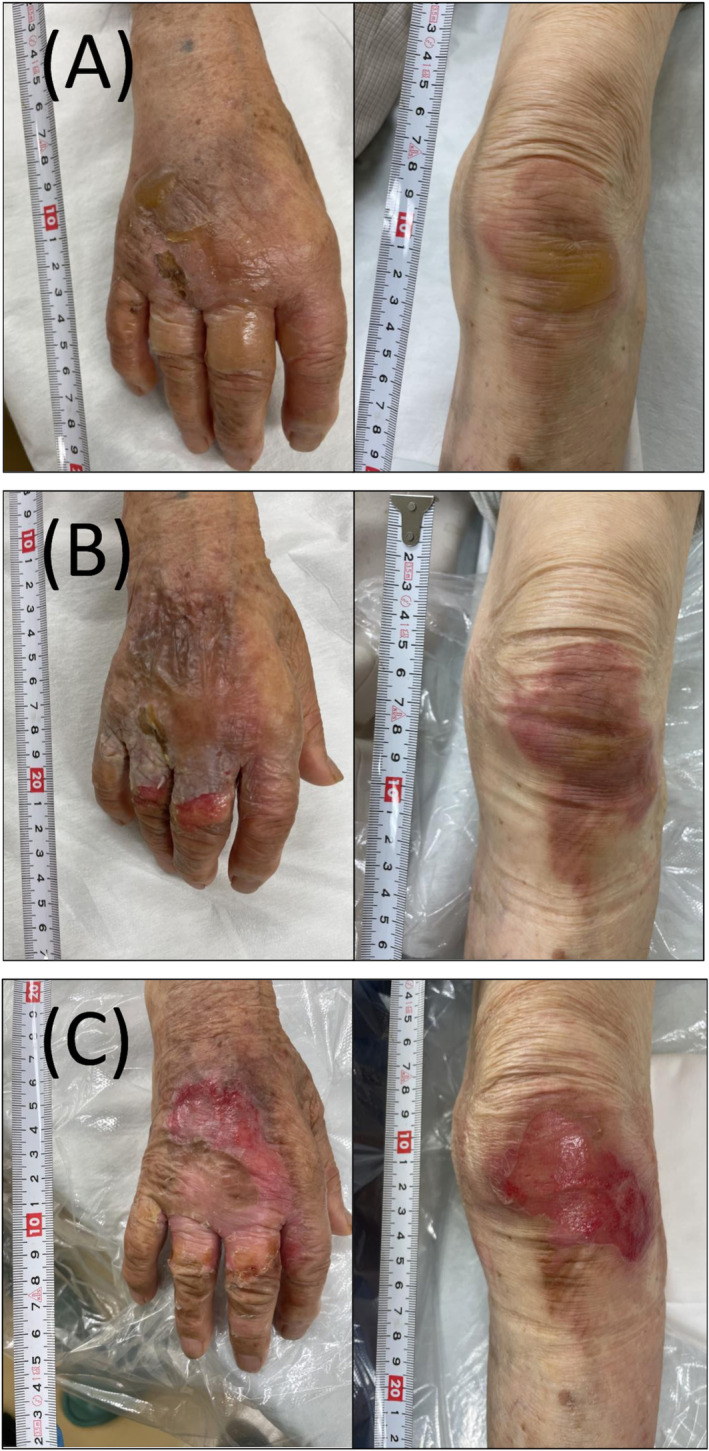
Burn injuries on the dorsum of his right hand and left knee ((A) at the day of the injuries, (B) after 3 days, (C) after 7 days).

**FIGURE 2:**
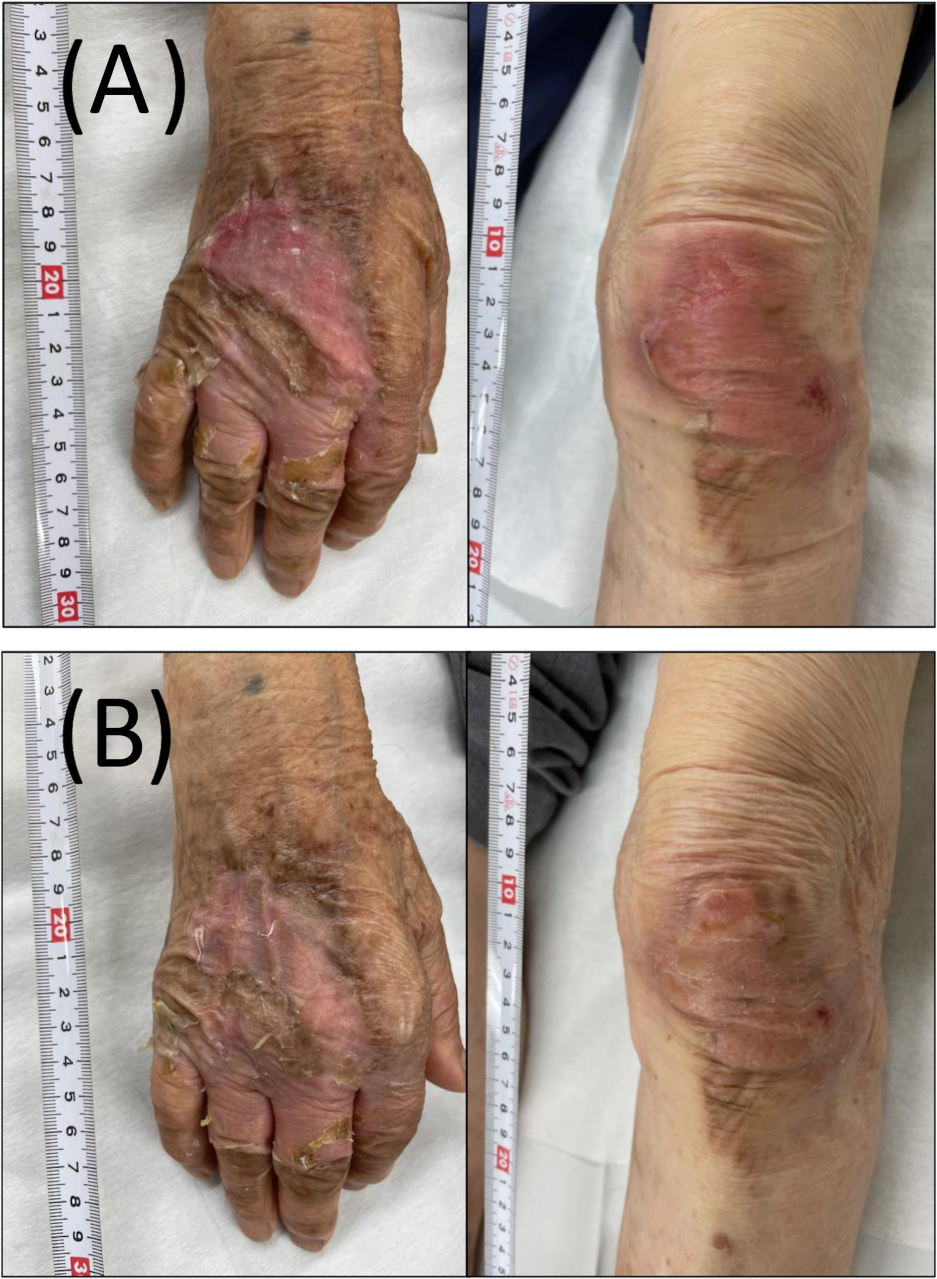
Burn injuries on the dorsum of his right hand and left knee ((A) after 10 days, (B) after 13 days).

A 96‐year‐old man who lived independently and had mild dementia, scoring 22 on the Mini Mental State Examination, sustained burn injuries from boiling water on the dorsum of his right hand and left knee. Both injuries were significantly painful, displaying redness, blisters, and edema after 3 h of the incident (Figure 1A). They were diagnosed as superficial partial‐thickness burns, and the burn wound bed area is approximately 2% of total body surface area (TBSA). He began taking 7.5 g/day of EPTJ extract (Tsumura & Co.) and applied Vaseline gauze dressing on the injuries, which was changed about every 3 days. He took 800 mg/day of acetaminophen to relieve pain and did not use any antibiotics or steroids. The blisters disappeared or self‐destructed within 3 days (Figure 1B). Redness and pain diminished after 7 days (Figure 1C). By Day 10, the pain had disappeared (Figure 2A), and by Day 13, the redness had subsided, and the injuries had epithelialized (Figure 2B). There were no side effects associated with EPTJ.

## 3 DISCUSSION

This case demonstrated that EPTJ has the potential to shorten the time to healing of superficial partial‐thickness burns. While there was a report of EPTJ being used for burns,^2^ this is the first report to employ it for a burn injury in an elderly patient, such as a 96‐year‐old man.

Eppikajutsuto possesses anti‐inflammatory effects and is also employed to reduce redundant body fluids, aiding in edema. Eppikajutsuto is extracted from six species of crude drugs: Mao (Ephedrae Herba), Sojutsu (Atractylodis Lanceae Rhizoma), Sekko (Gypsum Fibrosum), Shokyo (Zingiberis Rhizoma), Kanzo (Glycyrrhizae Radix), and Taiso (Ziziphi Fructus). The 7.5 g of EPTJ extract contains Ephedrae Herba 6.0 g, Atractylodis Lanceae Rhizoma 4.0 g, Gypsum Fibrosum 8.0 g, Zingiberis Rhizoma 1.0 g, Glycyrrhizae Radix 2.0 g, and Ziziphi Fructus 3.0 g respectively, provided by Tsumura & Co. Among these herbal medicines, Ephedrae Herba, Atractylodis Lanceae Rhizoma, and Gypsum Fibrosum are commonly found, and they are crucial components in the therapeutic effects of EPTJ. The exact mechanism has not yet been fully clarified, but Ephedrae Herba and Atractylodis Lanceae Rhizoma exhibit an anti‐inflammatory effect by reducing the production of inflammation‐related factors.^3^, ^4^ Gypsum Fibrosum is composed almost completely of CaSO4 and is known for relieving fever. Burns result in inflammation within a few hours of injury,^5^ and the inflammation and tissue edema of burn injuries can be resolved by using EPTJ.

According to a past research article, the healing time of a superficial burn wound with Vaseline gauze dressing is 9.30 ± 3.68 days in patients aged 18 to 65 years.^6^ Additionally, another article compared clinical outcomes in burn injuries with a median %TBSA ranging from 3.2 to 4.0 among different age categories: 18–64, 65–74, 75–85, and 85+ years. This article states that the median length of stay in the hospital almost tripled (*p* < 0.001) from 1.3 days per percent of TBSA in the younger category aged 18–64 years to 3.5 in the oldest one aged 85+.^7^ For these reasons, the healing time of a superficial burn wound in patients aged over 85 years old with Vaseline gauze dressing is expected to be much longer than 9.30 ± 3.68 days. The superficial burn injury in the case of the 96‐year‐old man subsided with Vaseline gauze dressing in 13 days, and there is a possibility that EPTJ could shorten the duration of healing. Moreover, the mortality rate was much higher in elderly cases than in younger ones, with a rate of 23.8 in the elderly category aged 85+ compared with 1.3 in the younger one aged 18–64 (*p* < 0.001).^7^

A past review indicates that the elderly people are at a higher risk of injury than younger age groups because of increased susceptibility. Even small, shallow burns are poorly tolerated by seniors, and two‐thirds of those who had been living independently before the burn were compelled to move into skilled nursing facilities following burn care. Dementia is also a significant risk factor for burns.^8^ The resolution of the burn injuries in this elderly patient within 2 weeks, despite having dementia, may suggest that EPTJ could have contributed to the treatment. After recovering from the injuries, he was able to continue living independently as before the injuries occurred.

Another past review revealed that geriatric burns are more common in developed countries than in developing countries. Furthermore, the proportion of elderly patients with burn injuries increased steadily from 1991 to 2005 in the United States. With the rapidly aging population, the number of geriatric burns is expected to increase.^9^ The findings of this case report will be valuable for primary care physicians because they will have many opportunities to treat elderly burn patients in the Japanese aging society in the coming years. This is the rare reported case in which EPTJ was used for burns, and further studies are warranted to clarify the benefits of EPTJ for burn injuries.

## 4 CONCLUSIONS

It is probable that EPTJ could be effective in reducing the healing time of superficial burn injuries, even in high‐risk patients such as the elderly people. Primary care physicians would find it helpful to keep in mind this treatment for burn injuries. However, Vaseline gauze dressing might be efficient in the relatively short duration of the wound in this patient. As few cases where EPTJ was used for burn injuries have been reported so far, further case reports or research are expected hereafter.

## CONFLICT OF INTEREST STATEMENT

Ryo Yoshinaga has received lecture fees from Tsumura & Co. No Conflict of Interest for other authors.

## ETHICS APPROVAL STATEMENT

None.

## PATIENT CONSENT STATEMENT

The author has obtained signed consent from the son of the patient authorizing publication.

## CLINICAL TRIAL REGISTRATION

None.
